# Temsirolimus, interferon alfa or both in advanced renal-cell carcinoma: One plus one does not always equal two

**Published:** 2007

**Authors:** S. Grover, J. C. Singh, N. S. Kekre

**Affiliations:** Department of Urology, Christian Medical College, Vellore, India. E-mail: chandrasingh@cmcvellore.ac.in

## SUMMARY

This Phase 3 multicenter randomized trial[1] compared interferon alone, temsirolimus alone or the combination of both for the treatment of newly diagnosed metastatic renal-cell carcinoma. From July 2003 to April 2005, a total of 626 patients were randomly assigned to one of the three study groups. Two hundred and seven were assigned to receive three million units of interferon alfa (with an increase to 18 million units subsequently) thrice weekly, 209 to receive 25mg of intravenous temsirolimus weekly and 210 to receive a combination of interferon and temsirolimus with 15mg of temsirolimus weekly plus six million units of interferon alfa thrice weekly. Those with histologically confirmed advanced renal-cell carcinoma (Stage IV or recurrent disease) and a Karnofsky performance score of 60 or more, with no previous systemic therapy and at least three of the six predictors of short survival were included. Patients were stratified according to the geographic location of the center and whether they had undergone nephrectomy. Treatment was continued as long as there was no disease progression, symptomatic deterioration or intolerable adverse events. Required imaging studies were done before treatment and were repeated at eight-week intervals to evaluate tumor size. The primary end point was overall survival, calculated on an intention-to-treat basis. This report was the second interim analysis conducted after 446 patients had died. Median survival was 7.3 months in the interferon group, 10.9 months in the temsirolimus group and 8.4 months in the combination therapy group and the median progression-free survival times in the interferon, temsirolimus and combination therapy groups were 1.9, 3.8 and 3.7 months, respectively. The objective response rates were 4.8%, 8.6% and 8.1% among patients receiving interferon, temsirolimus and combination therapy, not differing significantly. The effect of temsirolimus on overall survival was greater among patients under 65 years of age than among older patients and among patients with a serum lactate dehydrogenase level of more than 1.5 times the upper limit of the normal range than among those with lower levels.

## COMMENTS

Management of advanced renal-cell carcinoma (RCC) has made considerable progress in recent years and new emerging strategies are being developed. As distant metastases develop in about one-third of patients with RCC and most of these cases cannot be cured surgically, other options play an important role. Sunitinib, sorafenib and bevacluzimab have been proven to have efficacy in this scenario.[2] Both temsirolimus (CCI-779) and sirolimus (rapamycin), its primary metabolite, are potent and specific inhibitors of the mammalian target of rapamycin (mTOR) kinase, involved in intracellular signaling pathways of cell proliferation.[3] Interleukin-2 and interferon alfa, alone or in combination, have been the main treatments for metastatic renal-cell carcinoma. In select groups treatment with these agents results in a median survival of 12.0 to 17.5 months.[4] They rarely benefit patients with an extensive tumor burden and adverse prognostic factors. It was hoped that a combination of these agents may increase the degree of their antitumor effects.[5] In this Phase 3 trial, the principal finding was that, in patients with advanced renal-cell carcinoma and a poor prognosis, treatment with temsirolimus was associated with a moderate prolongation of overall survival than with interferon alone or the combination. The median overall survival in the group given temsirolimus alone was 10.9 months, as compared with 7.3 and 8.4 months in the groups given interferon alfa or combination therapy, respectively.

The combination of temsirolimus plus interferon did not improve overall survival. This could be due to greater adverse effects resulting in more delays and reductions in treatment and lower mean dose intensity of temsirolimus (10.9mg vs. 23.1mg per week). Patients with extensive and rapidly progressive disease may be less able to tolerate treatment and may have tumors that are inherently more resistant to therapy. Accordingly, the moderate efficacy of temsirolimus in advanced disease suggests that the drug might benefit patients with less extensive metastatic renal-cell carcinoma. The results of this trial point to mTOR as a target for cancer treatment and the possibility of using temsirolimus as first-line treatment for metastatic renal-cell carcinoma.

## References

[1] Hudes G, Carducci M, Tomczak P, Dutcher J, Figlin R, Kapoor A (2007). Temsirolimus, interferon alfa or both for advanced renal-cell carcinoma. N Engl J Med.

[2] Escudier B (2007). Advanced renal cell carcinoma: Current and emerging management strategies. Drugs.

[3] Raymond E, Alexandre J, Faivre S, Vera K, Materman E, Boni J (2004). Safety and pharmacokinetics of weekly intravenous infusion of CCI-779, a novel mTOR inhibitor, in patients with cancer. J Clin Oncol.

[4] Fyfe G, Fisher RI, Rosenberg SA, Sznol M, Parkinson DR, Louie AC (1995). Results of treatment of 255 patients with metastatic renal cell carcinoma who received high-dose recombinant interleukin-2 therapy. J Clin Oncol.

[5] Sosman JA, Puzanov I, Atkins MB (2007). Opportunities and obstacles to combination targeted therapy in renal cancer. Clin Cancer Res.

